# Representations of adult attachment and shame in parents of children on the autism spectrum

**DOI:** 10.3389/fpsyg.2025.1519090

**Published:** 2025-03-28

**Authors:** Charlotte Engberg Conrad, Marlene Briciet Lauritsen, Emil Færk, Helle Jakobsen, Per Hove Thomsen, Carol George

**Affiliations:** ^1^Psychiatry Unit, Aalborg University Hospital, Aalborg, Denmark; ^2^Department of Clinical Medicine, Aalborg University, Gistrup, Denmark; ^3^Department of Child and Adolescent Psychiatry, Psychiatry Unit, Aarhus University Hospital, Aarhus, Denmark; ^4^Department of Clinical Medicine, Aarhus University, Aarhus, Denmark; ^5^Department of Psychology, Mills College at Northeastern University, Oakland, CA, United States

**Keywords:** autism, adult attachment, parental attachment, shame, caregiving

## Abstract

Social communication disabilities in children on the autism spectrum challenge parenting. This is the first study to examine mental representations of adult attachment and shame in parents of children on the autism spectrum. Thirty-seven parents of children diagnosed with autism spectrum disorder (mean age 5.17 years) from middle to high-income households participated in the study. The Adult Attachment Projective Picture System was used to evaluate adult attachment patterns and representations as well as shame outcomes. All but three parents were classified as having *insecure* adult attachments. Almost half of the participants (45.9%) were classified as having *unresolved* attachments. All parents showed representations of a shamed self. Deep shame associated with attachment trauma was more common than normative shame. The sample was divided into *regulated* (*secure, dismissing,* and *preoccupied* combined) versus *unresolved* individuals. There were no significant group differences regarding shame or shame outcomes. The discussion addresses how the high frequency of *insecure* attachment representations and shame may affect parenting. This prevalence suggests that clinicians support families with children on the autism spectrum and introduce the topic of shame.

## 1 Introduction

Autism spectrum disorder (ASD) is a pervasive neurodevelopmental disorder characterized by disabled social interaction, communication, and repetitive stereotyped behaviors and interests (Organization WH, 1992). The prevalence of ASD is estimated to be 1–3% (Fombonne et al., 2021; Maenner, 2023). Being a parent of a child on the autism spectrum may be stressful and challenging. A central component of parenting is the intersection of biologically based behavioral systems of attachment and caregiving. These behavioral systems guide how dyads negotiate interactions surrounding the evolutionary function of the parent–child relationship to protect, care for, and comfort a child (George and Solomon, 2008; George and Solomon, 1996). Thus, autism may pose challenges to these caregiving goals. Previous research indicates shame is more common for parents of children on the autism spectrum than parents of children without autism (Marcinechová et al., 2023). The present study investigates the distribution of adult attachment, and the relationship between adult attachment and shame in parents of children with ASD.

Attachment is an innate neurobiological system that motivates the child to seek proximity to the primary caregiver for protection when stressed or feeling insecure (Bowlby, 1969). Attachment relations are developed in the interaction between the caregiver and younger child through dynamic communicative positive and negative interactions in daily life. Interactions are internalized as attachment representations (also termed internal working models (IWMs)) that are lifelong and influence how adults understand and interpret events and relationships (Bowlby, 1969; Bowlby, 1973; Bowlby, 1988). Research has shown that attachment patterns for most people are relatively stable over time from childhood to adulthood (Bowlby, 1969; Opie et al., 2021; Ainsworth et al., 1978) and can be transferred to the next generation (Stayton et al., 1971; Seskin et al., 2010; Buchheim et al., 2022; Van IJzendoorn, 1995; Benoit and Parker, 1994). However, both negative and positive events or relationships beyond childhood can influence the representations (Benoit and Parker, 1994; George et al., 1996).

The conceptualization of adult attachment was developed to correspond to child attachment. Following the child patterns, adult IWMs are classified into four patterns (George et al., 1996; Main and Solomon, 1986; Main et al., 1985). *Secure* attachment is characterized by emotional balance, confidence, and flexibility. There are two *regulated* patterns of attachment insecurity: one that creates distance and avoidance from stress or danger, and the other having anxious, enmeshed, and preoccupied defenses. *Unresolved* attachment is a dysregulated insecure pattern related to loss or trauma (George et al., 1996; Main and Solomon, 1986).

In attachment theory, parenting is organized by a behavioral system reciprocal to the child’s attachment behavior (Bowlby, 1969). Solomon and George (2021) elaborated on the framework of the caregiving system as being a behavioral system with an organized set of behaviors informed by the parent–child relationship and a complex transaction between concurrent experiential and ethological factors. It is essential to keep in mind that the purpose of the attachment system is to *seek* care and protection from an attachment figure, whereas the purpose of the caregiving system is to *provide* care and protection for a child (George and Solomon, 2008; George and Solomon, 1996). The desire to care for a child is biologically based (George and Solomon, 2008); however, caregiving behavior may be especially challenging for a child with disabilities in social communication. There are many challenges in parenting children on the autism spectrum that may put the caregiving system under attack and make parents feel helplessness and overwhelm (George and Solomon, 2008; Grey et al., 2021). These include for example, avoiding eye contact, overstimulation when held by the parent, the lack or deficiency of verbal language, and atypical non-verbal communication.

Early autism research pointed to the cause of autism as emotionally cold and distant mothers (Bowlby, 1969; Bettelheim, 1959; Kanner, 1943). This view was later rejected for the current view that autism is an innate neuro-biological disorder that is affected by the environment (Bölte et al., 2019; Bailey et al., 1996; Taylor et al., 2020; Elsabbagh, 2020). Historically, it was also thought that children on the autism spectrum were unable to form attachment bonds (Cibralic et al., 2018). This view has also been rejected. Systematic reviews of attachment and autism research show that 40–60% of children on the autism spectrum are secure (Kahane and El-Tahir, 2015; Rutgers et al., 2004; Teague et al., 2017). Like other children, children on the autism spectrum and secure attachment form bonds where the primary caregiver is sensitive to the child’s need for comfort (haven of safety) and exploration (secure base) (Ainsworth et al., 1978). Parenting sensitivity and effectiveness for a child on the autism spectrum may be impaired by difficulties in their children’s social communication (Cortina and Liotti, 2010; Stern, 2004). A higher frequency of insecure attachment in children on the autism spectrum seems not only related to both autism severity and cognitive development but also to more problematic parenting practices (e.g., coercion) (Teague et al., 2018).

Only a few studies have examined adult attachment representations in parents of children and on the autism spectrum (Seskin et al., 2010; Teague et al., 2017; Bond et al., 2020). One study assessed attachment representations using the Adult Attachment Interview (AAI) (George et al., 1996) within a sample of 40 parents (87.5% mothers, 12.5% fathers) (Seskin et al., 2010). Most of these parents had secure adult attachments; the classification distribution for secure and insecure parents of children on the autism spectrum was similar to a normative comparison sample (Seskin et al., 2010; Bakermans-Kranenburg and van IJzendoorn, 2009). This particular study demonstrated the beneficial developmental correlates of security; the children of *secure* parents had better relational and functional abilities than children of *insecure* parents (Seskin et al., 2010). Another study used a mixed-methods multiple case approach to investigate attachment representations with AAI in five parents. These parents had experienced a wide range of childhood adversities. As expected, all were classified *insecure* (Bond et al., 2020). More studies of this kind are needed (Teague et al., 2017). A study by Grey et al. (2021) explored the impact of parents’ attachment trauma upon caregiving of their children on the autism spectrum (Grey et al., 2021). Caregiving was measured using the Parent Development Interview (Aber et al., 1985). The study found that these parents had high levels of trauma from their early childhood, which shaped their caregiving strategies. Parents’ caregiving was driven by mal-adaptive, insecure, and shame-based defensive strategies of rejection, withdrawal and merging (Grey et al., 2021).

Shame is a social emotion developed in relation to other people and has recently been shown to be a companion in attachment and caregiving relationships (Grey et al., 2021; Lewis, 1987; Kaufman, 2004; Solomon and George, 2021). Shame is internalized as feelings of worthlessness, diminishment and exposure, as well as a need to escape or hide (Marcinechová et al., 2023; Tangney and Dearing, 2003). According to Cozolino (2017), shame is an emotional reflection of lost attunement with the caretaker that draws power from the child’s need to stay connected (Cozolino, 2017). It is intertwined with the child developing awareness of the self as an object in the eyes of a social partner during the first 18 months of life – developmental trajectory parallel to the consolidation of the attachment system (Erikson, 1994; Solomon, 2021). Schore (2015) model of interpersonal biology explains the overlap for the source attachment and shame in mother-infant right brain attunement. In essence, shame experiences systemically map onto and are an active contributor to the development of attachment (Solomon and George, 2021). Parents in many cultures use shame as part of normal parenting to guide the moral compass of wrong doing (Solomon and George, 2021; Schore, 2015). Shaming that goes beyond the boundaries of the moral compass is debilitating and can be held deeply as a facet of the internalized self-core. Deep debilitating shame creates feelings of helplessness, worthlessness, and abuse.

Functionalist emotion theorist Tomkins (1963) argues that emotional shame embodies constellations of self-evaluative emotions unified by the affective preoccupation with feelings of worthlessness. Specifically, people are hesitant to label their feelings as shame because they are uncomfortable with the term (Solomon and George, 2021; Tomkins, 1963). We point out here that a child’s diagnosis as “not typical” may produce feelings of shame in the parents. This phenomenon was reported in a study comparing 143 parents of children on the autism spectrum with 135 parents of children without autism. The parents of children on the autism spectrum experienced more shame than parents of children without autism. The qualitative analysis showed that shame was mostly related to the perception of the child’s inappropriate behavior or being misunderstood by society. Shame was also evidenced as a significant mediator of parental stress (Marcinechová et al., 2023). “If my child is wrong, I am wrong,” or “I have done something wrong. I am not a good parent.” Additionally, the symptoms of autism attracting attention in public settings may exacerbate a parent’s shame (Marcinechová et al., 2023; Grey et al., 2021; Conrad et al., 2024).

Crucial to understanding the role of shame in development and parenting is examining how the outcomes of shaming events. A caregiver’s ability to repair shame is the remedy for shame interactions. Schore (2015) argues that repaired shame re-establishes the relationship connection.

In attachment theory, acts of reparation are soothing and comforting, i.e., the essential elements of sensitive caregiving (Ainsworth et al., 1978). Repair reinstates positive affect and balance in the parent–child relationship (Solomon and George, 2021). Solomon (2021) suggested that shame outcomes are associated with children’s attachment patterns. It follows that the outcomes of parents’ representations of relationships when shamed would be associated with their adult attachment IWM.

This current study is the first to examine shame representations and outcomes of shame in the adult IWM of attachment in parents of children on the autism spectrum. The study also evaluates repair and other outcomes associated with the parent’s adult attachment representations.

### 1.1 Aims

The present study aims to investigate representations of adult attachment and shame and outcomes of shame in parents of children on the autism spectrum. Particularly, this study contributes to existing research by examining the following.

1. The attachment representation distribution in parents of children on the autism spectrum.
2. Differences in attachment-related shame between the *secure* versus *insecure* attachment or *regulated* versus *unresolved* attachment.
3. Differences in shame management between the *secure* versus *insecure* attachment or *regulated* versus *unresolved* attachment.

## 2 Materials and methods

### 2.1 Participants

This study is a cross-sectional investigation of parental adult attachment representations in a sample of mothers and fathers of children on the autism spectrum living in Denmark. Most participants were recruited from Danish clinical child and adolescent psychiatric departments and a Danish feasibility study of Paediatric Autism Communication Therapy (Conrad et al., 2024). One participant was recruited through social media. All participants received verbal and written information about the project before consenting to participation.

The sample is comprised of 37 parents of 24 children aged 3–7 years. Fourteen of the participants were parents who had participated in a national feasibility study of the parent-mediated intervention Paediatric Autism Communication Therapy after their child was diagnosed with ASD. The parents were geographically distributed across Denmark. Sixteen participants were parents of newly diagnosed children with ASD, as identified by the Department of Child and Adolescent Psychiatry, Aarhus (Denmark). Six participants were recruited while they were on the waiting list for assessment of a possible ASD in the Department of Child and Adolescent Psychiatry, Aalborg (Denmark). All children received a confirmed diagnosis of ASD. Twenty boys and four girls were included, and the mean age was 5.1 years (range 3.4–7.0 years). Subsequently, all children were diagnosed with ASD according to ICD-10 (F84.0, F84.1 and F84.5).

Five parents (13.5%) had self-reported mental disorders (personality disorder, OCD and depression, anxiety and depression, PTSD, and psychosis). Thirteen parents were heterosexual couples, and all were the biological parents of the child. The remaining 11 participants included three single parents and eight cohabiting parents. Of the eight cohabiting parents, all were living with the child’s other parent, who did not choose not to participate in the study. Participant demographics are shown in Table 1.

**Table 1:** Participant demographics.

Family characteristics *N* = 24		*N* (%)
Child living with	Both parents	20
	Mother	3
	Father	1
	Other	0
Language spoken in family	Danish	20
	Other	0
	Mixed	4
Household income in EUR	<26,800	0
	26,800–40,337	3
	40,337–67,230	4
	67,230–94,121	8
	<94,121–121,013	4
	121,013–161,351	3
	>161,351	2

Parent characteristics *N* = 37		
Age at birth of child		32.43 years
Recruited from:	Feasibility study	14
	Aarhus	16
	Aalborg	6
	Social media	1
Parents’ status	Mothers	22
	Fathers	15
	Other	0
Born in	Denmark	34
	Other	3
Psychiatric disorder	Yes	5 (13.5%)
	No	29 (78.4%)
	Do not know	3 (8.1%)
Education	Primary school (9–10 grade)	1
	Secondary school or short education	10
	Middle education (e.g., bachelor’s)	13
	Long education (e.g., master’s)	12
	PhD	1
Employment	Employed	33
	Unemployed	4
Time from diagnosis to AAP test	Mean when test after diagnosis (*N* = 31)	246.45 days
	Mean when test before diagnosis (*N* = 6)	266.67 days

Child characteristics *N* = 24		
Age at time of AAP test		5.17 years
Sex	Male	20 (83.3%)
	Female	4 (16.7%)
Born in	Denmark	24
	Other	0
Genetic Disorder	Yes	3
	No	21

In order to assess representability, the sample was compared to the population of mother and child dyads having children diagnosed with ASD in the Department of Child and Adolescent Psychiatry in Aarhus 2022. This population was chosen as a substantial part of our participants lived in this area.

### 2.2 Measures

Demographic information about child diagnosis, age, sex, ethnicity, parent educational and employment status, household income, and parent psychiatric diagnosis was obtained using self-report questionnaires. Information about diagnosis was obtained from the child’s clinical records.

Following Solomon and George (2021), we examined representations of shame and outcomes using a projective free response representational adult attachment assessment. According to Lewis (1987), shame is an emotion people have great difficulty articulating or acknowledging and is therefore best identified “in absentia” (Lewis, 1987). The Adult Attachment Projective Picture System (AAP) is a well-suited tool to evaluate shame in the context of attachment. Uncovering shame through a projective measure was expected to inform existing research on the presence and nature of shame in parents but without direct possibilities of explaining the sources of the shame.

#### 2.2.1 Adult attachment projective picture system

The assessment used in this study is a well-validated representational adult attachment measure, i.e., the AAP (George and West, 2011; Gander et al., 2017). The original validity study was based on an American/Canadian sample comprised of 144 participants aged 18–65. Validity was established by comparing classifications using the AAI and the AAP. The convergent validity between AAP and AAI for the four major attachment groups was 90% (kappa = 0.84, *p* < 0.000), and two groups (secure/insecure) classifications was 97% (kappa = 0.88, *p* < 0.000). Interrater reliability between two judges was 90% (kappa = 0.85, *p* = 0.00) for the four-group classification and 99% (kappa = 0.66, *p* = 0.000) for two-group classifications. Test–retest reliability after 3 months with 69 participants demonstrated an 84% (kappa = 0.78, *p* < 0.000) attachment classification agreement (George and West, 2012).

The AAP activates feelings and thoughts by asking participants to tell “stories” about the events and emotions evoked by pictures. All the pictures depict attachment events, including solitude, separation, illness, threat and death. Four depict settings where a person is alone, and three depict settings where the person is potentially in an attachment-caregiving relationship. The external stimuli of the pictures initiate an inner search for applicable mental concepts that come from perceptual and affective responses (George and Aikins, 2023). This method has been shown to be less intimidating and more likely to “surprise the unconscious” and right brain processes than an interview and challenges the validity of self-report measures monitored by defensive processes and the right brain (George and West, 2011).

The AAP identifies the standard four adult attachment patterns in the field: *secure, dismissing, preoccupied and unresolved* (George and West, 2011). Adults are judged *secure* when adults value attachment relationships, and their thinking about attachment and the self demonstrates integrated agency, emotional balance, confidence, and flexibility (George and West, 2011; George and Aikins, 2023). Adults are judged *dismissing* when they use deactivating defenses to create emotional distance from stressful situations and relationships. Bowlby (1980) explained how deactivation removes stress from conscious awareness (George and West, 2011; George and Aikins, 2023; Bowlby, 1980). Adults are judged *preoccupied* if they have an over-active attachment system fueled by defensive cognitive disconnection. Bowlby explained how disconnection splits and “fractures” access to affect and attachment information from their sources (George and Aikins, 2023; Bowlby, 1980). Deactivation and disconnection help individuals prevent stress from becoming overwhelming and are considered normative defenses (George and West, 2012). Finally, adults are judged *unresolved* when they are flooded by and cannot recovered from the emotional pain and overwhelm of loss or trauma (both of which are considered defense systems). Bowlby developed a third defense system, called *segregated systems,* as a contemporary form of repression. The flooding observed in *unresolved* individuals indicates chronic emotional vulnerability and a breakdown of normative organizing; flooded individuals cannot to contain or reorganize the feelings of being frightened and helpless that emerge in their stories (George and West, 2012; Bowlby, 1980). Research has shown a significant relationship between parents’ AAP representations and their children’s attachment as assessed in the Strange Situation procedure (Buchheim et al., 2022).

##### 2.2.1.1 AAP Administration

Validated as a virtual assessment, all AAPs were administrated virtually (David et al., 2022). Parents were instructed to use a tablet or computer to ensure a full view of the picture images. They were also instructed to be in a place where they would not be disturbed and asked not to eat or drink during AAP’s administration. Pictures were presented one at a time. Parents were instructed to describe what was happening in each picture, what led to the scene, what the people were thinking or feeling, and what would happen next. The assessment took between 15 and 30 min to complete. It was audio recorded and transcribed into verbatim transcripts.

The transcripts were coded according to the AAP manual standards by the first author, a trained, reliable Danish judge. Twenty percent of the transcripts were double-coded for reliability by another reliable Danish judge with 100% interjudge agreement.

##### 2.2.1.2 Shame coding

**Figure 1:**
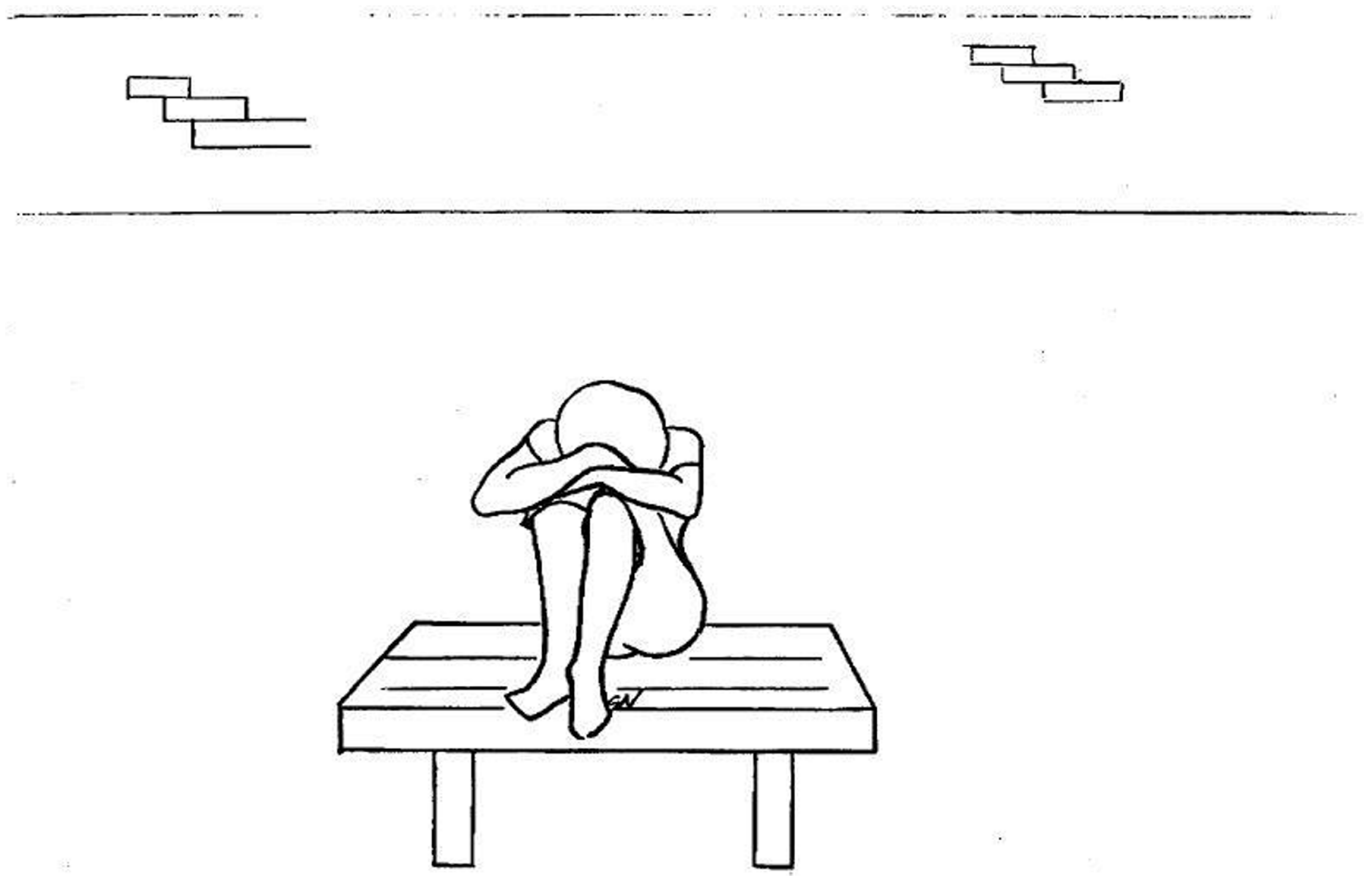
AAP picture stimulus “Bench”(© George et al., 1997–2025). Reproduced with permission from Carol George (© George et al., 1997–2025) (George and West, 2011).

**Figure 2:**
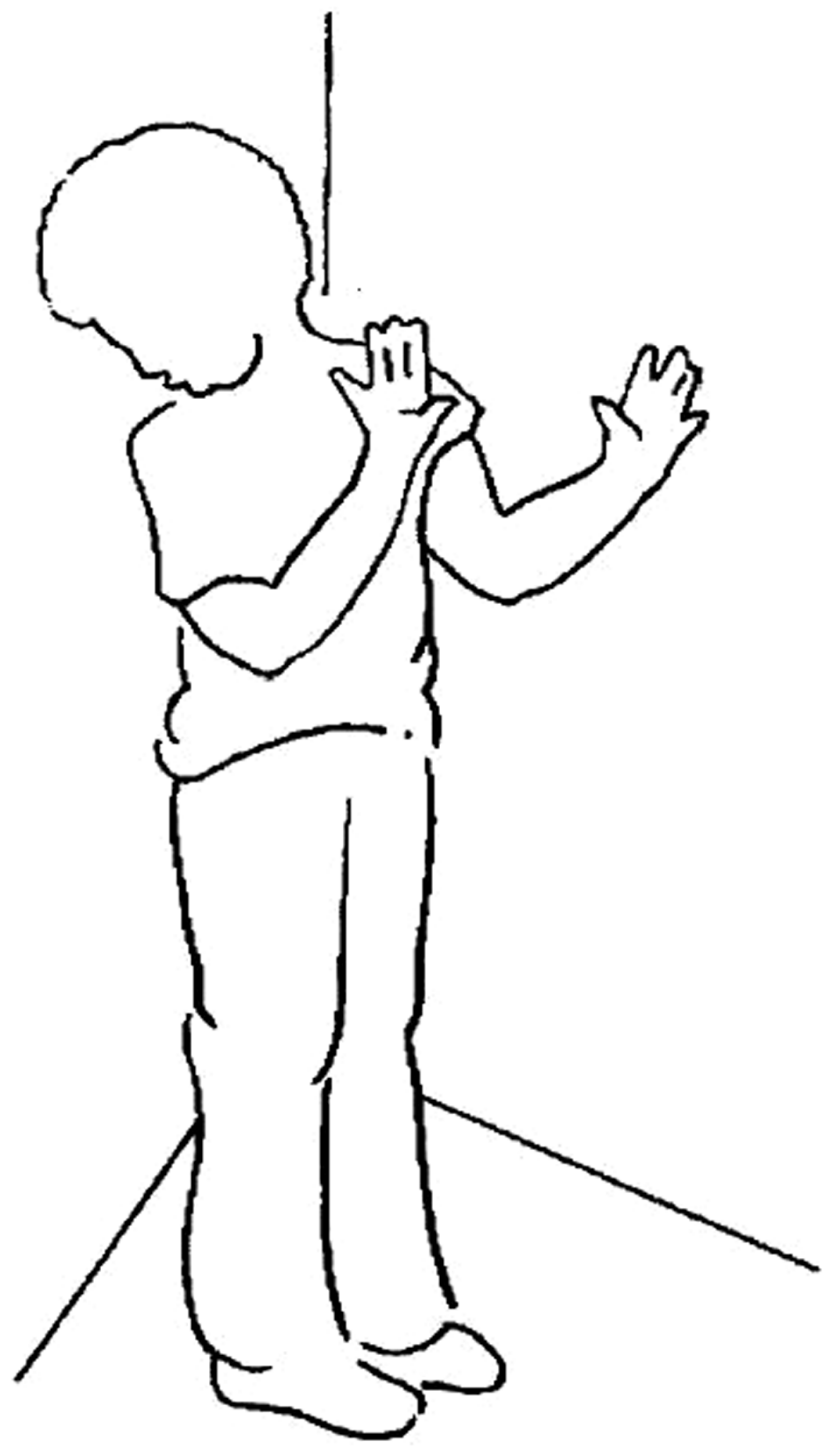
AAP picture stimulus “Corner”(© George et al., 1997–2025). Reproduced with permission from Carol George (© George et al., 1997–2025) (George and West, 2011).

Two AAP pictures known to elicit shame response were used in this study. One is an image of a person (ambiguous age and gender) sitting on a bench (i.e., Bench, Figure 1); the other is a child in a corner (i.e., Corner, Figure 2). Shame representations were designated as normative when the story narratives portrayed shame in response to typical conflict or distress related to socialization situations (e.g., the parent asked child to clean their room). Deep shame (popularly termed toxic shame) was identified when shaming events were traumatic, i.e., the main story character (the projected self) is described as feeling isolated, helpless, terrified, betrayed, or threatened. Six different types of shame were coded: behavior, affective preoccupation, core shame, traumatizing shame, shame defense, and hidden shame (Table 2). Behavior, affective preoccupation, and shame defense can apply to normative and deep shame. Core, traumatizing and hidden shame are forms of deep shame. The shame coding system is new and was developed during the work on this study by the last author CG. The system was informed by previous research in shame and attachment-related shame (Lewis, 1987; Kaufman, 2004; Kaufman, 1985) and use of clinical settings. The system was refined through the use in this study.

**Table 2:** Types of shame.

Type of shame	Definition
Behavior	Shame postures, e.g., hiding or wanting to become smaller
Affective preoccupation	Naming shame, depression, rumination, guilt, being disowned by others
Core shame	Shame self-appraisal of low self-worth, unjustified negative evaluation
Traumatizing shame	Helpless, isolated, threats to self, traumatic fear
Shame defense	e.g., contempt, withdraw, appease, shame-rage
Hidden shame	Shame hidden in games (e.g., hide and seek) or disavowing ordinary situations (e.g., shock in a water balloon fight)

Shame outcomes were designated as six different categories listed in Table 3. The stories were double coded authors CEC and CG. For this purpose, Bench and Corner stories were translated from Danish to English by CEC.

**Table 3:** Shame outcomes.

Shame outcome	Example
Integrated repair	The shamer is a member of an attachment-caregiving relationship and engages in integrative repair (i.e., sensitivity, uncompromised apology)
Instrumental reconciliation	The shamer in an attachment-caregiving relationship attempts to reconcile or restore the relationship without integrative repair
Functional management	The shamer or someone else focuses on practical remedies
Self-management	When the shamed self engages in self-protection
Incomplete or superficial	When ideas about repair or reconciliation are vague, fractured, or an attachment figure does not provide care
Unremedied	When there is no hope for an outcome

### 2.3 Data analysis

In statistical analysis, the categorical variable adult attachment representation is presented as frequencies. Following the attachment literature (JaPRS, 2016), the insecure groups are combined into a single group–*insecure* attachment. In a secondary analysis, the *secure, dismissing, and preoccupied* are combined into a single group of regulated attachment and compared with *unresolved* attachment. Chi-square or Fishers exact test (depending on sample size) is used to determine if group differences are significant in regard to representations of attachment and shame and the different outcomes of shame.

### 2.4 Ethical considerations

The study was reviewed by the local ethics committee which, according to Danish regulations, decided that no approval for the feasibility study was required. All data were kept in accordance with the European Union regulations, i.e., the General Data Protection Regulation. The study was registered at the regional research administration in the North Denmark Region (ID number F2022-050). Data used for assessment of representativity were derived after permission was given by the hospital in which the Department of Child and Adolescent Psychiatry in Aarhus is organized. Complete anonymity was ensured for the participants. Any names or quotes appearing in the transcripts and quotes were changed or removed to protect anonymity. Participation was voluntary, and participating parents were informed that their participation did not have any consequences for further treatment of their child. The interviewed participants all signed an informed consent form.

## 3 Results

### 3.1 Distribution of attachment representations

**Figure 3:**
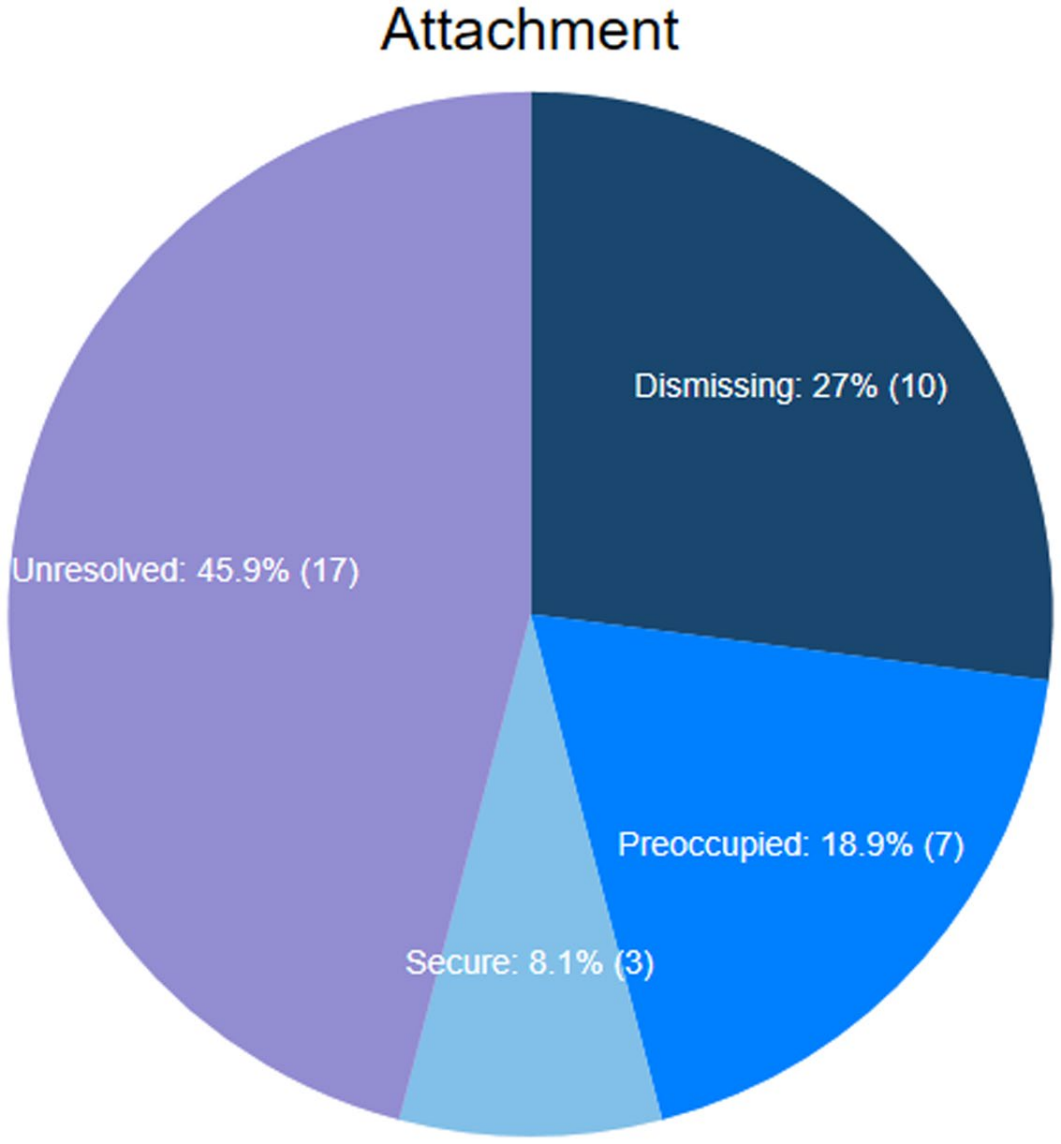
Distribution of adult attachment.

The sample attachment group distribution of 37 parents of children on the autism spectrum was *secure* 3 (8.1%), *dismissing* 10 (27.0%), *preoccupied* 7 (18.9%), and *unresolved* 17 (45.9%) (Figure 3).

**Figure 4:**
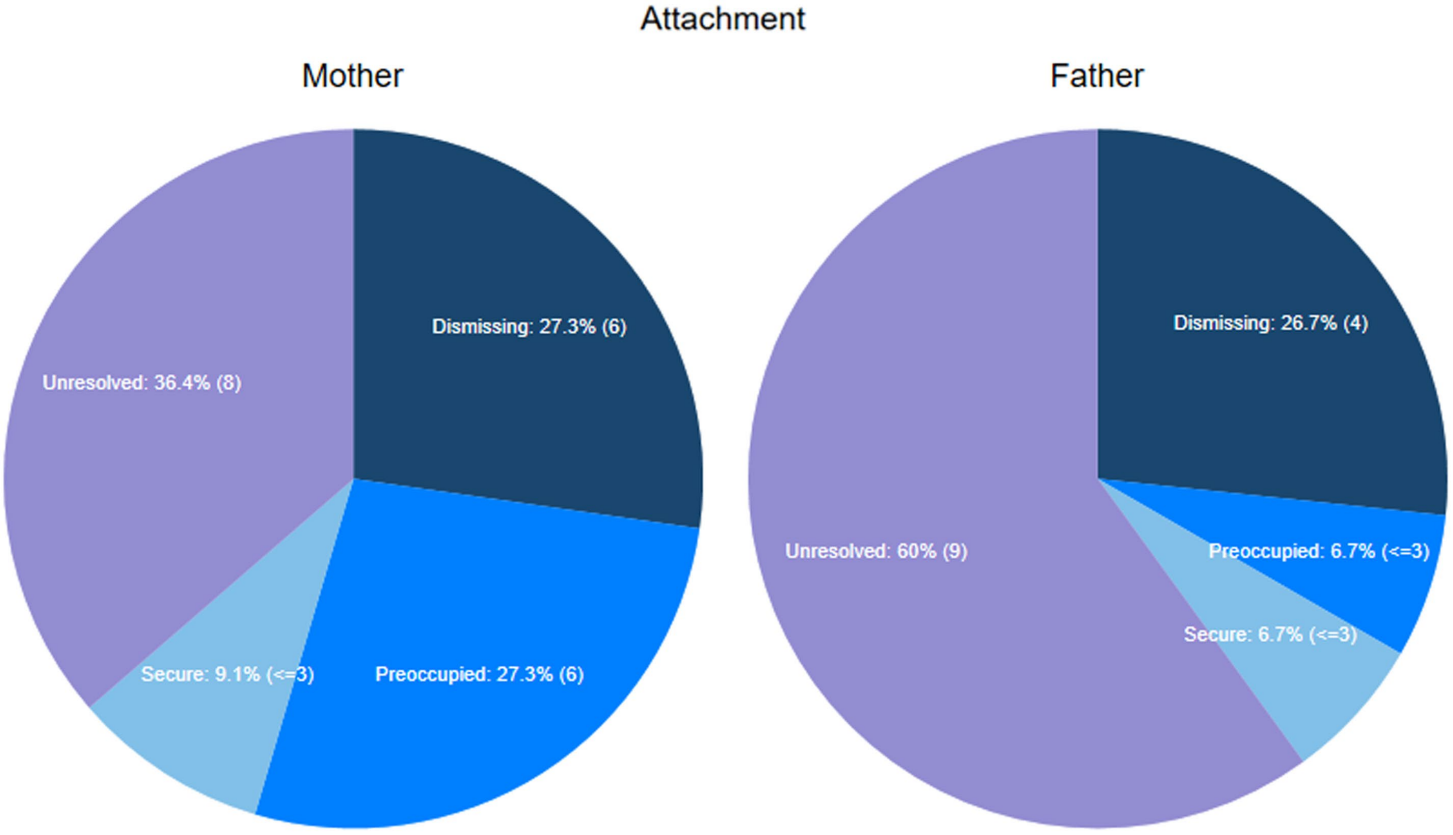
Distribution of adult attachment in mothers and fathers.

The proportions of *secure* and *dismissing* was similar for mothers and fathers. There was a higher number of *unresolved* fathers than mothers and a higher frequency of *preoccupied* mothers than fathers (Figure 4). A Fisher’s exact test, however, found no statistical significant differences between the parent groups (two-tailed *p* = 0.39).

The five individuals reporting having a mental disorder were all categorized insecure: Two with *dismissing*, one *preoccupied*, and two *unresolved*. The parents answering “Do not know” to the question of mental disorders were all classified *unresolved*.

Accessible data for 246 mothers from the Department of Child and Adolescent Psychiatry in Aarhus in 2022 showed a mean age at time of child’s birth of 30.81 years, which was only slightly younger than the age of both our mother and father participants. According to clinical records, 15.8% of the mothers had been diagnosed with a psychiatric disorder. Of the 246 children diagnosed with ASD, 201 (81.7%) were boys, and 45 (18.3%) were girls. Mean age at time of diagnosis was 5.01 years. There were no significant differences between groups regarding parents’ age (*t*-test, *p* = 0.36), children’s age (*t*-test, *p* = 0.23), parents with diagnosis (*X*^2^ = 0.50, *p* = 0.48), or children’s sex (*X*^2^ = 0.51, *p* = 0.48). Thus the sample was comparable to the clinical population of families having children diagnosed with ASD in the Department of Child and Adolescent Psychiatry in Aarhus in 2022 regarding age of mothers and children, as well as the frequency of parents having a psychiatric diagnosis, child age, and sex.

### 3.2 Shame in the AAP’s

Thirty seven participants were parents of children on the autism spectrum and all had AAP stories showing evidence of shame. Evidence of shame in only one story was found in only four participants.

There was no significant difference in the frequency of normative and deep shame in parents with *secure* versus *insecure* attachment representations, evaluated using Fisher’s exact test (two-tailed *p* = 0.61) (Table 4). This was not a robust comparison, however, as only three participants were judged *secure*. Both *secure* and *insecure* parents demonstrated deep shame. Although we expected the frequency of deep shame to be lower in *secure* individuals (66.7%) compared to insecure individuals (78.5%), the significance of this difference in this small sample could not be demonstrated. Overall, deep shame was more common than normative shame, which demonstrated a high level of trauma.

**Table 4:** Shame in parents with *secure* versus *insecure* IWM.

	Secure		Insecure		Total	
Shame	*N*	%	*N*	%	*N*	%
Normative	2	33.3	14	21.5	16	22.5
Deep	4	66.7	51	78.5	55	77.5
Total	6	100.0	65	100.0	71	100.0

Fisher’s exact test = 0.61.

The sample was divided into parents with *unresolved* versus *regulated* attachment. Fisher’s exact test found no statistical difference in the frequency of normative and deep shame in these groups (two-tailed *p* = 0.78) (Table 5). Similarly, there was no difference in this comparison between mothers and fathers (Fisher’s exact test two-tailed *p* = 1.00).

**Table 5:** Shame in parents with *Unresolved* versus *Regulated* IWM.

	**Unresolved**		**Regulated**		**Total**	
**Shame**	*N*	%	*N*	%	*N*	%
Normative	6	19.4	10	25.0	16	22.5
Deep	25	80.6	30	75.0	55	77.5
Total	31	100.0	40	100.0	71	100.0

Fisher’s exact test = 0.78.

When investigating the different kinds of shame, affective preoccupation was common within all attachment groups. Interestingly, core shame was only represented in the parents with *dismissing* IWM (3–30% of dismissing) and *unresolved* IWM (6, 35.3%). Shame defense was seen in all four groups: *secure* (2, 66.7%), *dismissing* (5, 50%), *preoccupied* (5, 71.4%), and *unresolved* (7, 41.2%).

### 3.3 Outcomes of shame

Shame outcomes were evaluated by examining the frequency of the different outcome strategies. We compared only the two groups of *regulated* and *unresolved* parents because of the small group size of *secure* parents, precluded a *secure* versus *insecure* comparison.

Contrary to our predictions, a Fisher’s exact test showed no significant outcome differences between *regulated* versus *unresolved* parents (two-tailed *p* = 0.18). Trend-level findings showed a higher frequency of functional repair in *regulated* parents and more unremedied repair in *unresolved parent*s (Table 6). Integrative repair was only seen in one story and as expected, this was in a *secure* parent.

**Table 6:** Outcomes of shame in parents with unresolved versus regulated IWM.

	Unresolved		Regulated		Total	
	*N*	%	*N*	%	*N*	%
Integrated repair	0	0.0	1	2.5	1	1.4
Instrumental reconciliation	5	16.7	3	7.5	8	11.4
Functional	2	6.7	8	20.0	10	14.3
Self-management	2	6.7	7	17.5	9	12.9
Incomplete	12	40.0	15	37.5	27	38.6
Unremedied	9	30.0	6	15.0	15	21.4
Total	30	100.0	40	100.0	70	100.0

Fisher’s exact = 0.18.

We also investigated any differences in shame outcomes in fathers versus mothers using Fisher’s exact test. There were no significant differences between the parent groups (two-tailed *p* = 0.90).

## 4 Discussion

The distribution of attachment representations in our sample of parents of children on the autism spectrum had a high frequency of parents with *unresolved* and *preoccupied* attachments. This sample had a slightly lower frequency of *dismissing* and fewer *secure* parents as compared with normative samples (Bakermans-Kranenburg and van IJzendoorn, 2009; Beetz et al., 2013). These findings contradict the results reported by Seskin et al. (2010), who found the attachment distribution in parents of children on the autism spectrum to be comparable with normative samples (Seskin et al., 2010; Bakermans-Kranenburg and van IJzendoorn, 2009). A high frequency of adults with *unresolved* attachment has been found in other clinical samples. In a study of 20 adults with intellectual disabilities, all were categorized as *insecure* using the AAP, with 60% with *unresolved* (Gallichan and George, 2018). Interestingly, a high frequency of *unresolved individuals* (41%) was also found in a control group of 17 healthy individuals compared to a group of 10 patients with borderline personality disorder (85% *unresolved*) (Buchheim et al., 2016). The control group was recruited through leaflets distributed in a hospital setting. This context suggested that the healthy controls could be related to someone with mental or physical illness, which is comparable to the parents in this study.

Only five parents reported being diagnosed with mental disorders; however, three parents reported “do not know” indicating having some mental issues. All parents with a known mental disorder were categorized with *insecure* IWM. The group of three suspecting mental disorders all had *unresolved* IWM. This frequency of mental disorders did not differ significantly from the frequency in the clinical sample of 246 mothers, and we have no reason to believe that our sample was not representative of parents with children on the autism spectrum.

The high frequency of unresolved attachment representations could also be because of a significant amount of trauma in several participants as revealed by the AAP. Roberts et al. (2013) found high levels of preexisting trauma (perinatal trauma and childhood abuse) in mothers of children on the autism spectrum compared to a normative sample (Roberts et al., 2013). Within the AAP coding, it is possible to distinguish individuals especially affected by attachment trauma. Individuals with *preoccupied* IWM characterized by the risk of chronic mourning are named “*preoccupied by personal suffering*.” Individuals with *dismissing* IWM with significant trauma in their AAPs typically fail to mourn and are identified as having *failed mourning* (George and Aikins, 2023). Within the group *preoccupied,* four out of seven participants qualified for *preoccupied by personal suffering*, and within the *dismissing* group, six out of ten individuals for *failed mourning*. This demonstrates that these parents are experiencing deep consequences of trauma and none of these parents would be considered regulated. When these groups with participants having significant amounts of trauma in their lives are combined with the group of regulated without trauma and parents who are secure as is standard in attachment research, these parents may not be comparable.

The differences in the distribution of attachment representations between fathers and mothers were not significant. This finding is consistent with previous findings of adult attachment (Bakermans-Kranenburg and van IJzendoorn, 2009). However, in the present underpowered sample nonsignificant differences were seen. Most remarkable were the differences in proportion of unresolved fathers (60%) as compared with mothers (36.4%). A previous study in this population indicated that there was a tendency of some fathers to adjust to the reality of the diagnosis later than the women (Conrad et al., 2024). Possibly, some of the men were more shocked and mentally shattered by their child’s diagnosis. Naturally, the reasons for their unresolved attachment would also be rooted in their childhood and the child’s diagnosis a trigger for continued flooding. Previous research shows that broader autism traits seen in family members of children on the autism spectrum are more prevalent in men – and it is possible that more men have been bullied because of these traits (Rubenstein and Chawla, 2018).

There is a notable risk of parental shame and stigma when their child is diagnosed with ASD (Hinshaw, 2005; Pianta et al., 1996; Bonis, 2016). In this study, some parents participated in the AAP while waiting for their child to be assessed for ASD, while others participated after the diagnosis. Previous research shows that parents of children on the autism spectrum are already more stressed before the diagnosis compared to other parents, as they are dealing with the discovery of the disabilities and delayed development of their child (Bonis, 2016; Silva and Schalock, 2012). Research shows that many parents are prepared and expect the diagnosis, but that receiving the diagnosis may still be accompanied by emotions of shock and grief (Conrad et al., 2024; Pianta et al., 1996). Hypothetically, some of the *unresolved* parents could be *unresolved* during this time of life, as they are feeling helpless and confused about how to adjust to the reality of their child’s diagnosis. Having a child with a disability like autism could be a crisis challenging the relative stability of the attachment system as defined by Bowlby and Ainsworth (Bowlby, 1969; Opie et al., 2021; Ainsworth et al., 1978).

Both representations of deep shame and normative shame were found in the parents regardless of their attachment classification. This study found all parents evidenced shame in their AAP stories. This finding is remarkable, given in proportion of parents’ shame stories in mothers from a community sample. That study reported shame in only 75% of the sample (Solomon and George, 2021). Our data confirm a trend of *regulated* parents as having more efficient strategies for managing shame than the *unresolved* individuals. Functional repair was more frequent in the *regulated* group of parents, while unremedied repair was more frequent in the group of parents with *unresolved* parents. Our the trend-level findings indicate some association with attachment status may be found in a larger sample equipped to compare *secure* and *insecure* groups. This thinking is strengthened by the fact that integrated repair was only identified in a *secure* individual. However, this picture was not as black and white in reality as it was in theory. For example, a few *unresolved* parents endorsed an instrumental reconciliation as a strategy. Also, unremedied repair was seen in all *insecure* groups of attachment and not only in the *unresolved* parents. In this study, none of the six stories from *secure* parents showed *unremedied* repair, which was as expected. If the sample had been larger, we could have compared the secure and insecure groups of parents and possibly demonstrated differences in shame outcomes between the groups. The findings confirmed that there were no differences in shame variables between fathers and mothers.

The finding of core shame identity only being present in *dismissing* and *unresolved* parents is consistent with attachment theory. *Dismissing* adults may use their deactivating defenses to create emotional distance and remove shame from conscious awareness (Bowlby, 1980). As with other negative emotions, deactivating defenses bury feelings of unworthiness. Also, as expected, the construct of core shame identity was associated with *unresolved* attachment*. Unresolved* parents may be flooded by emotional pain and overwhelmed by trauma and feelings of failure and unworthiness associated with shame. Instead of burying it like *dismissing* parents, *unresolved* parents cannot regulate being overwhelmed by their shamed self. Additionally, they may have been subject to attachment traumas such as abdication of caregiving by their own parents (George and Solomon, 2008; George and Aikins, 2023). These parents may feel inadequate in their caregiving of their child on the autism spectrum and unable to contain or reorganize their story (Solomon, 2021).

The question is how shame and *insecure* adult attachment affect parenting (i.e.,the caregiving system). According to previous research, *insecure* parents use caregiving strategies that foster insecurity in their children, such as functional care without comfort, rejecting emotions, heightened non-contingent care, or helplessness with abdication of care (George and Solomon, 2008; George and Aikins, 2023). The result of a recent qualitative study with parents of children on the autism spectrum is consistent with this thinking; it confirmed that these parents were help-seeking and appreciative of the support they could get (Conrad et al., 2024). This study found that a developmental intervention the Paediatric Autism Communication Therapy seemed to have an effect of reducing shame in parents as their parental reflective functioning and sensitivity in caregiving is enhanced (Conrad et al., 2024). It is likely that other developmental and attachment based interventions will have the same effect. The finding of a high frequency of *insecure* parents in this sample highlights the need for supporting families with children diagnosed with ASD. Also, the finding of all participating parents having shame representations, mostly deep shame, demonstrated that this is an aspect of parenting that has been neglected. As clinicians, we need to talk about shame with the parents during the process of having their child diagnosed with ASD.

It is established that AAP is about childhood experiences (George and West, 2011). Attachment is a lifespan concept, influenced by events after childhood, including the birth of an atypical child. The parents, in some instances, referred to difficult and even shameful or traumatic situations they currently experienced with their child on the autism spectrum. In some instances, they directly mentioned their child on autism spectrum. More often, the stories concerned parents not being able to understand their children, children being “not normal,” or children acting out of control in response to everyday activities.

The Bench stories were often concerning children being disowned by others. This could be both representations of the parents’ childhood memories and representations of their child on the autism spectrum experiencing being disowned by others. A previous study confirms that parents of children on the autism spectrum worry their children would get bullied or victimized more than other parents worry (Ashburner et al., 2019). Linking to the theories of the Broader Autism Phenotype, recognizing how some parents of children on the autism spectrum carry autism traits – such as pragmatic and communication difficulties, poor social skills, rigidity, stereotyped behaviors, impaired emotional recognition, and aloofness (Bolton et al., 1994; Gerdts and Bernier, 2011). It seems likely that these parents during their lifetime could have been disowned and bullied by others. The shame representations could be a reflection of something that had happened or their fear that this would happen since their child was not like most children. The AAP cannot only evoke childhood attachment relationships but also current attachment relationships.

Previous research has demonstrated how *unresolved* parents tend to merge psychologically with their children and may have difficulties distinguishing between their own and their children’s experiences (George and Solomon, 2008). Theoretically, these possible implications of autism on the parents’ attachment representation will affect the caregiving system, leaving parents helpless, broken, and in need of help to rebalance their caregiving strategies towards their child (George and Solomon, 2008). The Corner story is sometimes told in the AAP with direct references to children on the autism spectrum. Some of the parents told stories about a child overwhelmed by sensory stimuli or refusing everyday activities like eating or brushing teeth, which would be common for children on the autism spectrum. The pictures for parents can likely activate stories from everyday activities originating from both their own and their children’s childhood.

### 4.1 Strengths and limitations

The strengths of this study were that it is one of the first to explore attachment patterns in parents of children on the autism spectrum, and two, it is the first to explore shame representations in the parents. Our study adds knowledge to a growing body of exploring attachment and shame representations in mothers and fathers. Although developmental and attachment research tends to emphasize mothering, mothers and fathers both contribute importantly to children’s socialization. This is especially the case in European-based families.

Another strength of the study is that we were able to obtain data about the hospital-based population of parents having children aged 2–6 years old diagnosed with ASD in Aarhus in 2022. This data indicated that our sample was representative of the general population of parents with ASD. However, the data was limited to mothers, age, sex and diagnosis and no information regarding, e.g., income or ethnicity, could be obtained.

The study was limited by small sample size. Recruiting participants for this research project within families with younger children on the autism spectrum proved considerably harder than expected. Several families did not want to participate due to the considerable strain (and probably shame) of getting their child diagnosed. Participating in the study would have added a burden to the busyness of everyday lives with younger children. As a result, it was not possible to reliably compare *shame* outcomes in parents with *secure* versus *insecure* parents.

Another limitation is that the shame research using the AAP is new and only partly validated. The constructs of shame in the AAP draw on extensive research on shame, and during the development of the shame coding system, CG validated representations of shame seen in their AAPs with clients (Solomon and George, 2021). More validation studies are needed. Additionally, the choice of a projective measure to assess representations of shame, leads to a lack of clarity as to the source of the shame.

## 5 Conclusion

This was the first study to examine representations of adult attachment and shame in parents of children on the autism spectrum. The findings revealed a high frequency of *unresolved* and *insecure* adult attachment in the parents, which was not as expected. However, this high frequencies of unresolved individuals is comparable to studies connected to hospital settings. The parents’ high level of trauma could be due to experiences of disownment and trauma from their childhood, and their worry and perceptions about their child on the autism spectrum. These findings expand our view on how we understand and interpret the AAP in future research and clinical settings.

We did not find evidence of associations between shame representations or outcomes and attachment representations. Still, the trend-level findings in this small sample of possible associations between attachment classifications and shame repair call for more research on this topic using larger samples. More research investigating adult attachment representations and shame in parents of children on the autism spectrum is needed.

Our findings show that many parents of children on the autism spectrum in this sample experience traumatizing shame. Thus, we recommend that clinicians working with families with children on the autism spectrum increase their efforts to provide emotional and practical support for both parents and children. We additionally want to highlight how traumatizing deep shame is an aspect that is present in parents and how clinicians could help by talking about shame.

## Data Availability

The raw data supporting the conclusions of this article will be made available by the authors, without undue reservation.
